# ITC-6102RO, a novel B7-H3 antibody-drug conjugate, exhibits potent therapeutic effects against B7-H3 expressing solid tumors

**DOI:** 10.1186/s12935-023-02991-x

**Published:** 2023-08-18

**Authors:** Seol Hwa Shin, Eun Jin Ju, Jin Park, Eun Jung Ko, Mi Ri Kwon, Hye Won Lee, Ga Won Son, Yun-Yong Park, Yeon Joo Kim, Si Yeol Song, Sangkwang Lee, Beom Seok Seo, Jin-A Song, Sangbin Lim, Doohwan Jung, Sunyoung Kim, Hyangsook Lee, Seok Soon Park, Seong-Yun Jeong, Eun Kyung Choi

**Affiliations:** 1 Asan Medical Institute of Convergence Science and Technology, Asan Medical Center, University of Ulsan College of Medicine, Seoul, 05505 Republic of Korea; 2 Asan Institute for Life Sciences, ASAN Medical Center, Seoul, 05505 Republic of Korea; 3 Asan Preclinical Evaluation Center for Cancer Therapeutics, ASAN Medical Center, Seoul, 05505 Republic of Korea; 4 Department of Life Science, Chung-Ang University, Seoul, 06974 Republic of Korea; 5 Department of Radiation Oncology, ASAN Medical Center, University of Ulsan College of Medicine, Seoul, 05505 Republic of Korea; 6 IntoCell Inc, 101, Sinildong-ro, Daedeok-gu, Daejeon, 34324 Republic of Korea

**Keywords:** Antibody-drug conjugate (ADC), B7-H3, Ortho hydroxy-protected aryl sulfate (OHPAS)-linkers, Patient-derived xenograft (PDX)

## Abstract

**Background:**

The B7-H3 protein, encoded by the CD276 gene, is a member of the B7 family of proteins and a transmembrane glycoprotein. It is highly expressed in various solid tumors, such as lung and breast cancer, and has been associated with limited expression in normal tissues and poor clinical outcomes across different malignancies. Additionally, B7-H3 plays a crucial role in anticancer immune responses. Antibody-drug conjugates (ADCs) are a promising therapeutic modality, utilizing antibodies targeting tumor antigens to selectively and effectively deliver potent cytotoxic agents to tumors.

**Methods:**

In this study, we demonstrate the potential of a novel B7-H3-targeting ADC, ITC-6102RO, for B7-H3-targeted therapy. ITC-6102RO was developed and conjugated with dHBD, a soluble derivative of pyrrolobenzodiazepine (PBD), using Ortho Hydroxy-Protected Aryl Sulfate (OHPAS) linkers with high biostability. We assessed the cytotoxicity and internalization of ITC-6102RO in B7-H3 overexpressing cell lines in vitro and evaluated its anticancer efficacy and mode of action in B7-H3 overexpressing cell-derived and patient-derived xenograft models in vivo.

**Results:**

ITC-6102RO inhibited cell viability in B7-H3-positive lung and breast cancer cell lines, inducing cell cycle arrest in the S phase, DNA damage, and apoptosis in vitro. The binding activity and selectivity of ITC-6102RO with B7-H3 were comparable to those of the unconjugated anti-B7-H3 antibody. Furthermore, ITC-6102RO proved effective in B7-H3-positive JIMT-1 subcutaneously xenografted mice and exhibited a potent antitumor effect on B7-H3-positive lung cancer patient-derived xenograft (PDX) models. The mode of action, including S phase arrest and DNA damage induced by dHBD, was confirmed in JIMT-1 tumor tissues.

**Conclusions:**

Our preclinical data indicate that ITC-6102RO is a promising therapeutic agent for B7-H3-targeted therapy. Moreover, we anticipate that OHPAS linkers will serve as a valuable platform for developing novel ADCs targeting a wide range of targets.

**Supplementary Information:**

The online version contains supplementary material available at 10.1186/s12935-023-02991-x.

## Background

Lung cancer is among the most frequently diagnosed malignancies in terms of both incidence and mortality rates [1]. A majority of patients with lung cancer are diagnosed at advanced stages of the disease, which contributes to a poor prognosis and a 5-year survival rate of less than 20% [2]. Conventional treatment modalities for lung cancer include surgical resection, chemotherapy, radiation therapy, and combination therapies [3]. However, these approaches are often limited by the severe side effects associated with surgery and radiation [4, 5]. Breast cancer is the most commonly diagnosed cancer in women and the second leading cause of cancer-related death among this population [6, 7]. While cure rates for early-stage breast cancer continue to improve, metastatic breast cancer remains largely incurable, despite recent advancements in treatment outcomes [8]. Current research focuses on the development of a new generation of anticancer agents that target specific molecular markers. One such example is the human epidermal growth factor receptor 2 (HER2), which has been identified as a key molecular target in breast cancer patients. Several HER2-targeting agents have been approved by the United States Food and Drug Administration (FDA) for clinical use [9, 10]. Although these agents have demonstrated efficacy in HER2-positive breast cancer patients [11], more than 70% of HER2-non-positive patients have not benefited from HER2-targeted therapies. Consequently, there remains a critical unmet need for effective targeting agents to address this therapeutic gap in the treatment landscape.

B7-H3 (B7 homolog 3 protein), alternatively known as CD276 or B7RP-2, is a transmembrane glycoprotein belonging to the B7 ligand family, which plays a critical role in antitumor immune responses [12, 13]. This protein is highly expressed in tumor cells across diverse cancer types, with approximately 60% of all patients exhibiting high-frequency expression. Overexpression of B7-H3 has been associated with tumor progression, metastasis, and poor clinical outcomes in various malignancies [14–16]. In contrast, B7-H3 expression is limited in normal tissues [15, 17], sparking considerable interest in evaluating B7-H3 as a biomarker for targeted therapy.

Antibody-drug conjugates (ADCs) are a class of cancer therapeutics consisting of a monoclonal antibody, linkers, and a toxic payload (toxin), each of which is crucial for ADC efficiency and safety [18, 19]. ADCs enable the selective delivery of cytotoxic drugs to targeted tumor tissues, thereby enhancing the therapeutic window by selectively administering cytotoxic drugs to target cells [20]. Typically, the chosen toxin induces apoptosis via microtubule inhibition or DNA damage, subsequently eliminating surrounding cancer cells through the bystander effect [21]. Moreover, the choice of linker and toxin significantly influences the stability and efficacy of ADCs [22]. Mylotarg (gemtuzumab ozogamicin), the first ADC drug, was approved by the United States FDA in 2000. Over the past two decades, 14 ADCs have been introduced to the market, reflecting a growing trend in ADC clinical development [23]. Unfortunately, while three B7-H3 ADCs are currently undergoing clinical trials, none have received approval.

Previous research demonstrated that the microtubule-disrupting agent monomethyl auristatin F (MMAF) can be conjugated to an anti-B7-H3 monoclonal antibody using two different linker types: the Ortho Hydroxy-Protected Aryl Sulfate (OHPAS) linker and the valine-citrulline-p-aminobenzyloxycarbonyl (VC-PABC) linker [24–26]. The resulting ADC with the OHPAS linker exhibited stability in mouse and IgG-depleted human plasma, while the ADC with the VC-PABC linker displayed instability in mouse plasma [24]. This study aimed to develop a novel B7-H3-ADC, evaluate its antitumor effects, and demonstrate B7-H3 as a promising therapeutic target for solid tumors overexpressing B7-H3. Consequently, a novel B7-H3-ADC (ITC-6102RO) was generated utilizing the highly stable OHPAS linker, as previously confirmed, and dHBD (heterocycle-fused benzodiazepine dimer), which offers enhanced solubility compared to pyrrolobenzodiazepine (PBD). The preclinical efficacy of ITC-6102RO for solid tumors was substantiated through in vitro and in vivo analyses.

## Methods

### Cell lines and culture

Human lung NCI-H23 cells (American Type Culture Collection, ATCC no. CRL-5800, Manassas, VA) were cultured in Roswell Park Memorial Institute (RPMI) 1640 medium (Gibco-Invitrogen, Carlsbad, CA). Human breast carcinoma JIMT-1 cells (Deutsche Sammlung von Mikroorganismen und Zellkulturen GmbH, DSMZ no. ACC 589, Inhoffenstraße, Braunschweig) were maintained in Dulbecco’s Modified Eagle Medium (DMEM) (Gibco-Invitrogen). Both media were supplemented with 10% fetal bovine serum (Gibco-Invitrogen) and 1% penicillin/streptomycin (Gibco-Invitrogen), and the cells were incubated in a humidified atmosphere containing 5% CO_2_ at 37 °C. Mycoplasma contamination was assessed using a MycoAlert PLUS Mycoplasma Detection Kit (Lonza Walkersville, Inc., Walkersville, MD).

### Antibodies

Antibodies to detect B7-H3 (ab134161) and PARP-1(ab32071) were from Abcam. Alpha-tubulin (#2125), cyclin A (#4656), p-Chk1 (#2341), and Chk1 (#2345) antibodies were bought from Cell Signaling Technology. Anti-beta-actin (A5441) was bought from Sigma-Aldrich. Cyclin B1 (MA5-13128) and Cyclin D1 (MA5-12702) antibodies were bought from Thermo Fisher Scientific. Cyclin E (18–0404) antibody was from Zymed Laboratories. Caspase-3 (ADI-AAP-113) antibody was obtained from Enzo. Anti-gamma-H2AX (A300-081 A-9) was acquired from Bethyl Laboratories, Inc. Anti-rabbit IgG horseradish peroxidase (706-036-148), mouse IgG, (711-036-152) and goat IgG (705-036-147) were bought from Jackson ImmunoReserch.

### Generation of ITC-6102RO

**Fig. 1:**
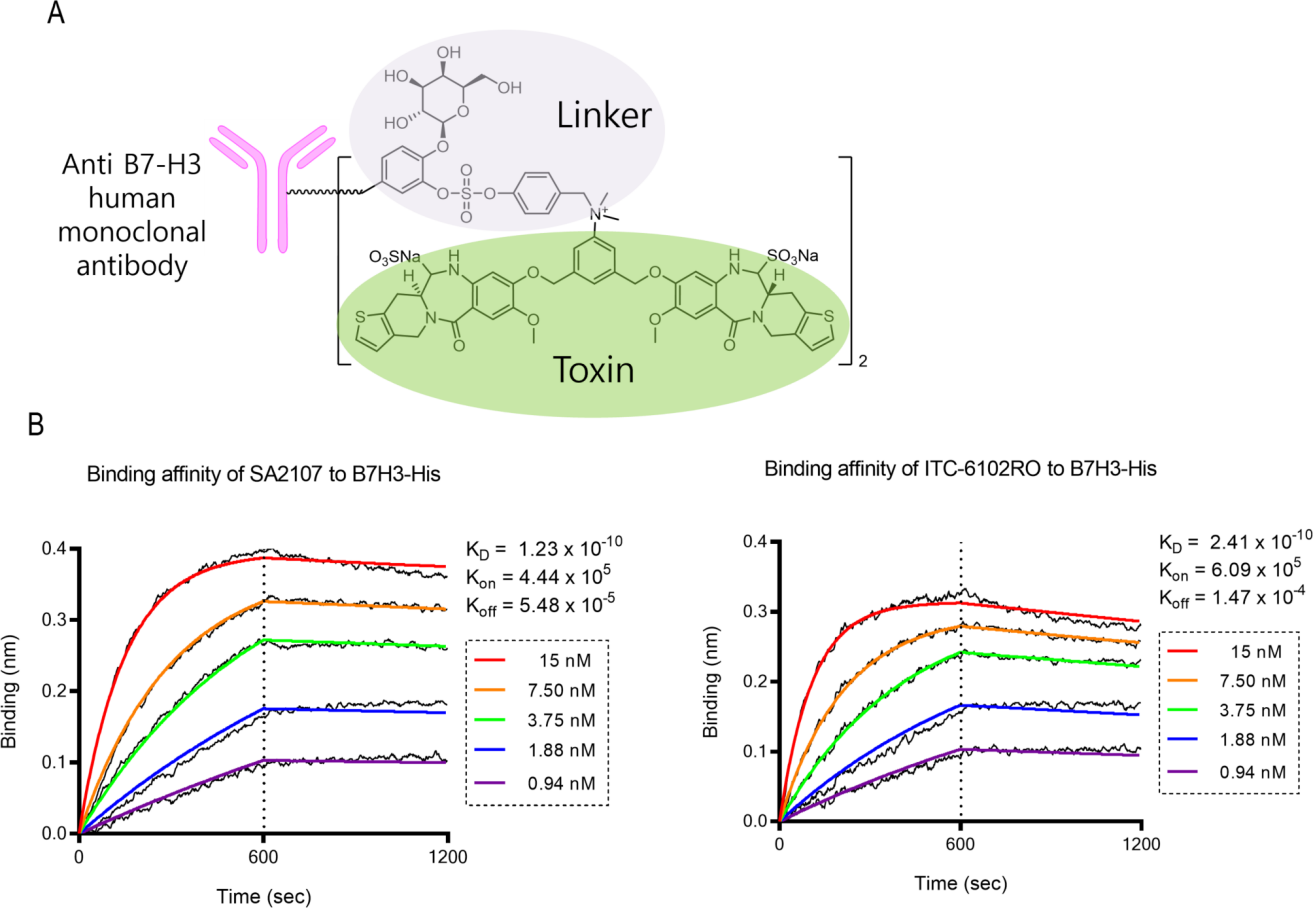
Characteristics of ITC-6102RO. (**A**) Composition of the ADC. (**B**) Binding affinity between antibody and ADC. BLI kinetic assays were performed using an Octet QKE biosensor by first capturing 10-µg/ml antibodies with anti-human Fc (AHC) biosensors, followed by a 300 s baseline step in kinetic buffer. Binding sensorgrams were obtained using the 8-channel detection mode on the Octet QKE unit

The structure of ITC-6102RO is presented in Fig. 1A. ITC-6102RO is a B7-H3-targeting ADC and comprises an anti-B7-H3 antibody and a benzodiazepine dimer derivative, which is a DNA cross-linking agent, connected by a maleimide-containing OHPAS linker. The linker-payload and antibody-drug conjugate was prepared at IntoCell. Thiomab site-specific conjugation technology was used for ITC-6102RO. Prior to conjugating the B7-H3 thiomab to dHBD via the maleimide-containing OHPAS linker, any blocking cysteine or glutathione present on the thiomab was eliminated by mild reduction in PBS at 37 °C through the addition of a 5000-fold molar excess of reducing agent L-cysteine, followed by diafiltration. To re-establish the inter-chain disulfide bond, the reduced thiomab was incubated for 24 h at 4 °C. A 4.5-fold molar excess of maleimide-linker drug was then incubated with the activated thiomab for one hour at 40 °C, followed by diafiltration. Hydrolysis of the succinimide ring in the linker drug occurred under 50 mM borate buffer (pH 9.2) for 1.5 h at 37 °C. The antibody conjugate was subsequently purified using preparative HIC column chromatography, and the number of conjugated linker drugs per antibody was quantified through HIC-HPLC analysis.

### Binding affinity and binding selectivity

The BLI kinetic assays were performed using an Octet QKE biosensor (ForteBio, Fremont, CA) by first capturing 10 µg/mL antibodies with anti-human Fc (AHC) biosensors, followed by a 300-second baseline step in a kinetic buffer. The antibody-captured biosensors were then immersed in wells containing varying concentrations of B7-H3 antigen for 600 s, followed by a 600-second dissociation step in the kinetic buffer. The kinetic buffer used in the baseline, association, and dissociation steps was consistent across all processes. Binding sensorgrams were obtained utilizing the 8-channel detection mode on the Octet QKE instrument. Fresh AHC biosensors were employed without any regeneration steps.

### Flow cytometric analysis

In this study, flow cytometry was employed to analyze various cell lines, including NCI-H23, JIMT-1, Calu-6, NCI-H358, and Jurkat. Adherent cells were washed twice with PBS and subsequently detached using Accutase (Sigma) at 37 °C. The cell suspension was transferred to a 50 mL conical tube, and FACS buffer (PBS containing 2% FBS) was added in a 2:1 v/v ratio (FACS buffer:cells). Cells were pelleted by centrifugation at 250×g and resuspended in FACS buffer at 4 °C. A total of 5.0 × 10^5^ cells/well were dispensed into 96-well u-bottom plates (Costar, Cole-Parmer Canada Company, Montreal, CA). Pre-diluted antibody samples were added to the wells to achieve a final volume of 100 µl/well and concentrations ranging from 0 to 14 nM (5-point dilution series), followed by a 30-minute incubation at 4 °C. Cells were subsequently washed twice by centrifugation at 250×g, with supernatant removal by aspiration, and resuspension in 200 µl FACS buffer at 4 °C. Detection reagent, anti-human Fc AlexaFluor488, was added, and samples were incubated at 4 °C for 20 min. Cells were washed twice in 200 µl FACS buffer, and flow cytometric analysis was performed on a CytoFLEX instrument (Beckman Coulter, Brea, CA). Median fluorescence intensity was determined for 20,000 live cells per sample.

### Internalization analysis

Calu-6 cells were seeded onto 12-mm poly-L-lysine-coated cover glass in six-well plates. Cells were stained with 10 µg/ml of SA2107 and ITC-6102RO for one hour at 4 °C. Following two washes with cold PBS, cells were incubated at 37 °C for the indicated time intervals in growth medium containing 10 mM NH_4_Cl. Cells were fixed using 4% paraformaldehyde (PFA) in PBS, and residual PFA was quenched with PBS. Subsequently, cells were permeabilized using a staining buffer composed of 0.5% saponin and 10% FBS in PBS. Incubation with the LAMP-1 marker antibody, fluorophore-conjugated secondary antibody, and washing steps were conducted in the staining buffer. Samples were mounted with DAPI, and cells were imaged using a Zeiss LSM800 confocal microscope (Zeiss, Oberkochen, Germany).

### In vivo rat pharmacokinetics of ITC-6102RO

The preclinical PK studies were performed using male SD rats. ITC-6102RO was administered as a single intravenous bolus injection at a dose of 3 mg/kg. Blood samples were collected at 0, 0.002, 0.042, 0.167, 0.29, 1, 2, 4, 7, 14, 21, and 28 days post-injection into heparinized tubes. These samples were centrifuged, and the resulting plasma supernatants were stored at -80 °C until further analysis. Plasma concentrations of ITC-6102RO (quantified as an antibody-drug conjugate via cleavable linker enzyme digestion), total antibody (including both conjugated and unconjugated antibodies), and dHBD (free payload) were determined using LC-qTOF-MS. Below the lower limit of quantification (BLQ) for ITC-6102RO and total antibody was < 0.100 mg/mL, while for dHBD, it was < 0.100 µg/mL. Pharmacokinetic parameters were calculated employing non-compartmental analysis and a two-compartment model using Phoenix WinNonLin® version 8.0.0 (Certara, Princeton, NJ, USA).

### Cell viability assay

Exponentially growing H23 and JIMT-1 cells were harvested and seeded in 96-well plates at a density of 7 × 10³ cells/well in 100 µL growth medium. Each experiment was performed in triplicate. Cells were exposed to anti-B7-H3 monoclonal antibody (Naked mAb), ITC-02-050 (free drug), or ITC-6102RO at concentrations ranging from 0.001 to 1 µM for 48 h. Cell proliferation was assessed using the CCK-8 assay kit (Dojindo Molecular Technologies, Gaithersburg, MD, USA) according to the manufacturer’s instructions.

### Western blot analysis

Cells were washed in PBS and lysed in boiling sodium dodecyl sulfate/polyacrylamide gel electrophoresis (SDS-PAGE) sample buffer (62.5 mM Tris, pH 6.8, 1% SDS, 10% glycerol, and 5% beta-mercaptoethanol). The lysates were boiled for five minutes, resolved by SDS-PAGE, and transferred to an Immobilon membrane (Millipore). Nonspecific binding sites were blocked for one hour using 5% skim milk, after which the membranes were incubated for two hours with specific primary antibodies. Membranes were subsequently washed three times with TBST and incubated for an additional hour with horseradish peroxidase-conjugated anti-rabbit, mouse, or goat secondary antibodies. Protein bands were visualized using enhanced chemiluminescence (ECL, Amersham Life Science) and imaged with ImageQuant LAS 4000 (GE Healthcare Life Sciences).

### Annexin V/PI staining

To perform Annexin V/propidium iodide (PI) staining, cells were first digested with trypsin and washed twice with PBS. The staining was carried out in accordance with the Annexin-V-FLUOS Staining Kit (Roche) instructions. Cells were stained with 5 µl of Annexin-V-FLUOS and 1 µl of PI staining solution, protected from light, at room temperature for 15 min. Flow cytometric analysis of cell samples was conducted using a Becton-Dickinson instrument.

### Cell cycle distribution

H23 and JIMT-1 cells were seeded in a 60-mm tissue culture dish at a density of 5 × 10^5^ cells/plate and incubated overnight. The cells were treated with either anti-B7-H3 monoclonal antibody (Naked mAb), ITC-02-050 (free drug), or ITC-6102RO at a concentration of 0.1 µM for 48 h. Post-treatment, cells were harvested, suspended in ice-cold 100% ethanol for fixation, and subsequently stained with PI. Flow cytometry using a BD FACSCanto II instrument was employed to analyze the cell cycle distribution.

### Immunohistochemistry (IHC)

Tumor tissue samples from mice were fixed in 4% paraformaldehyde overnight and permeabilized with 70% ethanol for an additional overnight period. Paraffin-embedded tumor Sect. (3 μm) were mounted onto silane-coated slides. The tissue was blocked with 5% bovine serum albumin (NGS) in PBST (0.01% Triton X-100 in PBS) and incubated with primary antibodies diluted in PBST at 4 °C overnight. Following primary antibody incubation, tissue sections were incubated with horseradish peroxidase-conjugated secondary antibodies for one hour at room temperature. Visualization was achieved using diaminobenzidine (DAB, Vector SK 4100). Tissue sections were examined under a DP71 microscope (Olympus, Tokyo, Japan) after counterstaining with hematoxylin.

### In vivo tumor growth delay

For the evaluation of in vivo therapeutic efficacy, JIMT-1 CDX and PDX tumor models were established using male athymic nude mice (BALB/c-nude, 6 weeks old; Japan Shizuoka Laboratory Center, Hamamatsu, Japan). A suspension of 5 × 10^6^ cells for the JIMT-1 CDX model or a 3 × 3 × 3 mm^3^ tissue block for the PDX model was subcutaneously implanted into the right hind leg of each mouse. Mice with xenograft tumors reaching 80–120 mm^3^ were pair-matched based on tumor volume and assigned to experimental and control groups (n = 5 per group). Drug administration for the JIMT-1 CDX model began 10 days post-cell transplantation, whereas for the lung PDX models, 11C38 and 15C63, initiation of treatment was at 12 days and 41 and 48 days post-tissue transplantation, respectively. PBS and ITC-6102RO were intravenously (IV) administered via the tail vein at single doses of 0.15, 0.3, or 0.6 mg/kg. Tumor volumes and body weights were monitored throughout the study, and tumor volumes were calculated using the formula: volume = (length × width^2^) × 0.5. The results are presented as the mean ± standard deviation.

### Statistical analyses

Sample sizes were not predetermined, and all data are presented as the mean ± standard deviation (SD) from at least three independent experiments. Means were compared using a two-tailed t-test, and *p*-values were obtained using Microsoft Excel 365. P-values are denoted as *p < 0.05, **p < 0.01, or ***p < 0.001, indicating significant differences.

### Study approval

All animal experiments adhered to a protocol approved by the Institutional Animal Care and Use Committee of the Asan Institute for Life Science (2019-12-334, 2020-12-026). Signed informed consent was obtained from all patients. The study received approval from the Institutional Review Board of Asan Medical Center (2010–0618).

## Results

### Expression profile of B7-H3 in patient tumor tissues

**Fig. 2:**
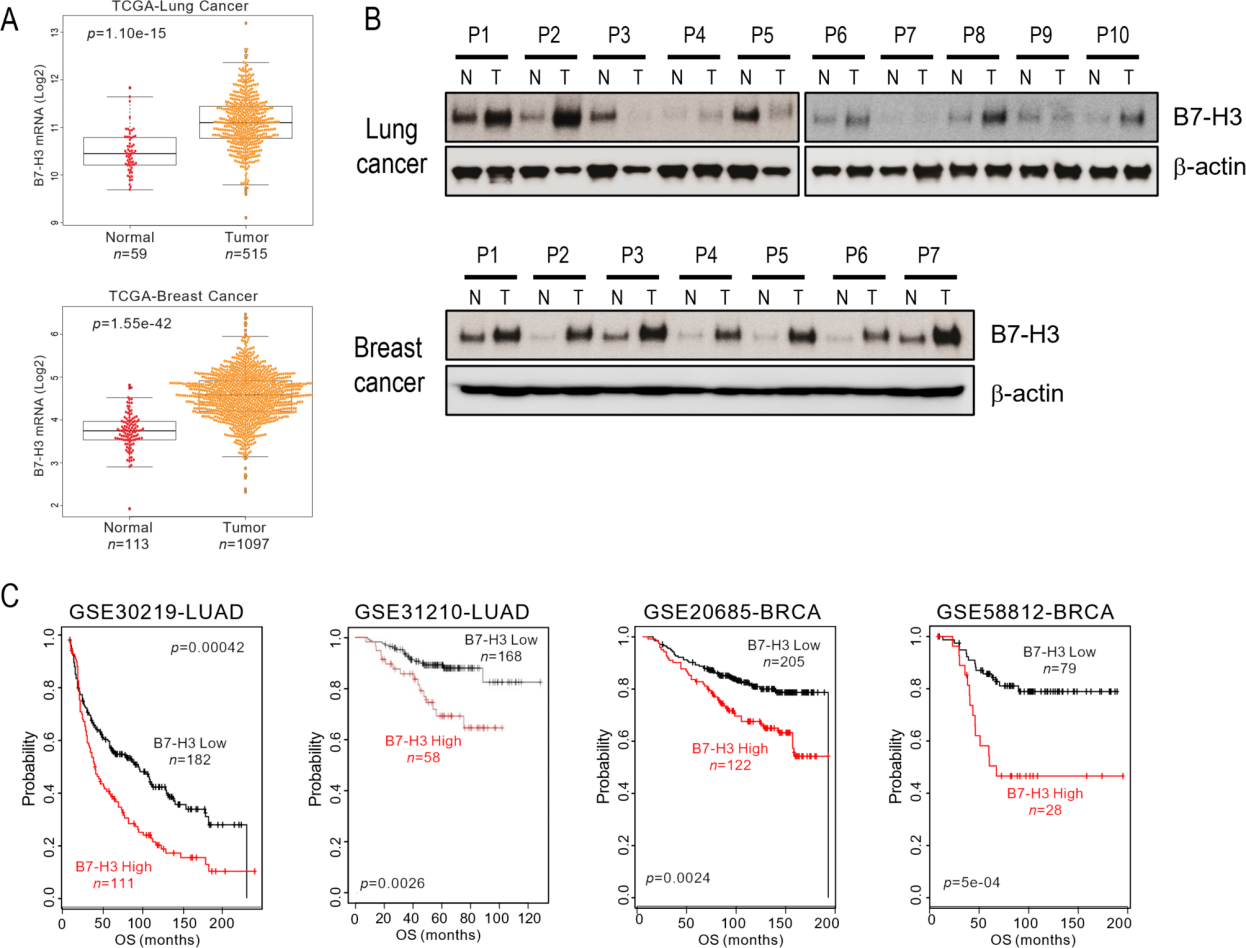
B7-H3 expression in lung and breast cancer. (**A**) B7-H3 mRNA levels in lung and breast cancer patient cohorts from the TCGA database. (**B**) Western blotting of B7-H3 expression in tumor and normal tissues of lung and breast cancer patients. Equal loading of protein samples was verified using anti-β-actin. (**C**) Patients in lung and breast cancer cohorts were divided into groups with relatively high or low B7-H3 expression for the Kaplan-Meier plot

Our analysis revealed that B7-H3 expression was consistently detected in separate lung and breast cancer cohorts, indicating its prominent presence in tumor samples (Fig. 2A). A comparative assessment showed elevated B7-H3 protein expression in lung and breast cancer tissues relative to normal tissues (Fig. 2B). Further examination of lung and breast cancer datasets demonstrated a significant association between B7-H3 expression and patient survival (Fig. 2C), implying the potential of B7-H3 as a biomarker for targeted therapy. Collectively, these findings suggest that B7-H3 expression is upregulated in lung and breast cancer and exhibits a strong correlation with unfavorable patient outcomes.

### A novel B7-H3-ADC, ITC-6102RO is generated with an OHPAS-linker technology and show an improved pharmacokinetic profile

**Fig. 3:**
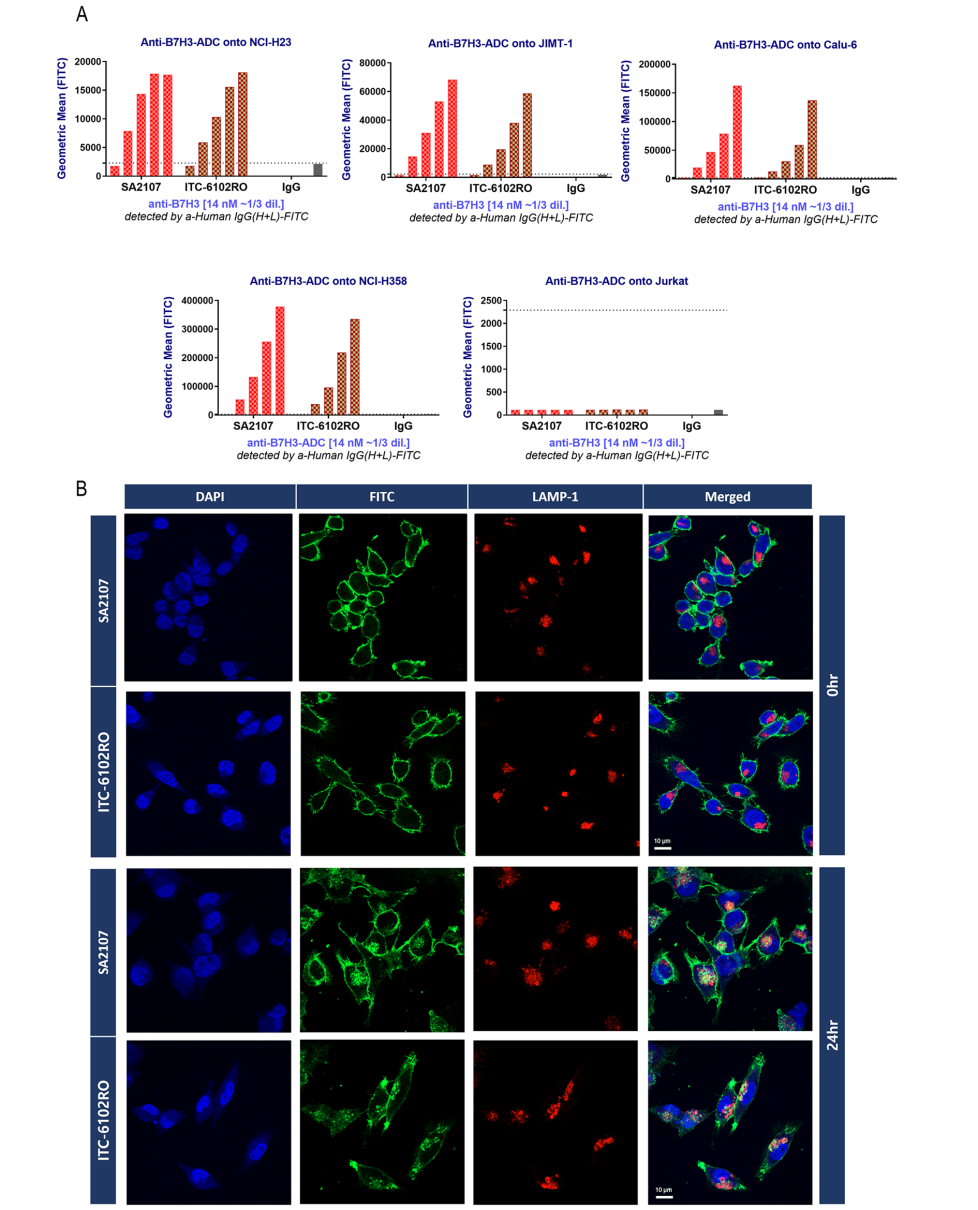
Internalization of ITC-6102RO. (**A**) Binding selectivity in cell lines. Cells were seeded at 5.0 × 10^5^ cells/well in 96-well u-bottom plates. Pre-diluted antibody samples were added to reach a final volume of 100 µl/well and concentrations ranging from 0–14 nM (5-point dilution series), followed by incubation at 4 °C for 30 min. The detection reagent, anti-human Fc AlexaFluor488, was added and incubated at 4 °C for 20 min. Flow cytometric analysis was performed using CytoFLEX (Beckman Coulter, Brea, CA). (**B**) Internalization of ITC-6102RO by anti-B7-H7 antibody in Calu-6 cells. Calu-6 cells were seeded on 12-mm poly-L-lysine-coated cover glass in six-well plates. Cells were stained with 10 µg/ml of SA2107 and ITC-6102RO for one hour at 4 °C. Cells were fixed in 4% paraformaldehyde (PFA) in PBS and permeabilized with staining buffer (0.5% saponin, 10% FBS in PBS). LAMP-1 marker antibody and fluorophore-conjugated secondary antibody incubations, as well as wash steps, were performed in the staining buffer. Samples were mounted with DAPI and imaged using a Zeiss LSM800 confocal microscope (Zeiss, Oberkochen, Germany)

As previously reported, the OHPAS linker represents a novel di-aryl sulfate structure featuring a phenolic payload and a self-immolative group. It exhibited stability in both in vitro mouse/human plasma and in vivo pharmacokinetic studies using mice. In the present study, a novel B7-H3 ADC, ITC-6102RO, incorporating the OHPAS linker was employed. Anti-B7-H3 mAb (SA2107, Naked mAb) was conjugated to the dHBD (heterocycle-fused benzodiazepine dimer) via the beta-galactosidase-sensitive, cleavable OHPAS linker (Fig. 1A). The drug-antibody ratio (DAR) of ITC-6102RO was approximately 2.0. HIC-HPLC analysis revealed that dHBD conjugation did not influence the antibody-binding activity (toxin-unconjugated anti-B7-H3 mAb (SA2107): KD = 1.23 × 10^− 10^ nM; ITC-6102RO (dHBD-conjugated anti-B7-H3-ADC): KD = 2.41 × 10^− 10^ nM; Fig. 1B). The binding selectivity of ITC-6102RO was assessed using BLI kinetic assays in B7-H3-positive cell lines (NCI-H23, JIMT-1, Calu-6, and NCI-H358) and the B7-H3-negative cell line, Jurkat. Cell surface-bound anti-B7-H3 was quantified by flow cytometry after exposure to ITC-6102RO, using FITC-labeled anti-human IgG H + L. As depicted in Fig. 3A, anti-B7-H3 was detected in a dose-dependent manner in B7-H3-positive cell lines, whereas B7-H3-negative Jurkat cells showed no anti-B7-H3 binding. These findings suggest that the binding of ITC-6102RO is dependent on B7-H3 expression.

The internalization of ITC-6102-RO in the B7-H3-positive Calu-6 cell line was assessed via immunofluorescence analysis. Comparable internalization of SA2107 and ITC-6102RO was observed in Calu-6 cells (Fig. 3B). To verify ITC-6102RO translocation to lysosomes, further immunofluorescence analysis was conducted. Upon incubation of SA2107 or ITC-6102RO-exposed cells at 4 °C for one hour, ITC-6102RO membrane staining diminished, and ITC-6102RO localization within lysosomes was evident through the colocalization of FITC and the lysosomal marker LAMP-1 (Fig. 3B). A similar pattern was observed for the toxin-unconjugated SA2107. These findings indicate that ITC-6102RO initially binds to the membrane of B7-H3-expressing cells before being internalized and translocated to the lysosomal compartment at 24 h.

**Fig. 4:**
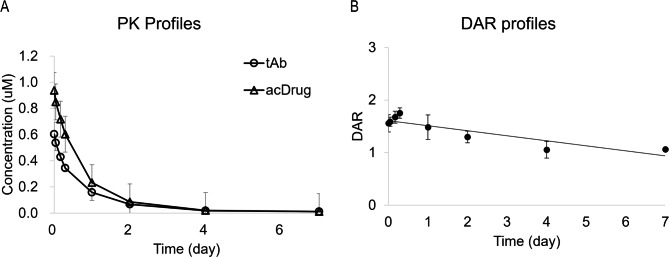
PK profiles of ITC-6102RO. (**A**) In rats, the pharmacokinetic profiles of tAb (total antibody) and acDrug (antibody-conjugated drug) after intravenous administration of 3 mg/kg ITC-6102RO. (**B**) The drug-antibody ratio (DAR) profiles in rat plasma for ITC-6102RO. The number of conjugated linker drugs per antibody was quantified using HIC-HPLC analysis

The pharmacokinetic (PK) profile of ITC-6102RO was examined in rats and found to be comparable to that of the B7-H3 monoclonal antibody. Enhanced biostability of ITC-6102RO relative to the B7-H3 monoclonal antibody was observed in rat plasma up to day two (Fig. 4A). Furthermore, stable attachments of the linker-toxin (drug-antibody ratio; DAR) were detected in rat plasma (Fig. 4B) up to day seven. These findings demonstrate that ITC-6102RO was engineered for a more precise DAR, exhibiting improved biostability in rat serum compared to the B7-H3 monoclonal antibody.

### ITC-6102RO successfully inhibited the proliferation and induced apoptosis of B7-H3-expressing cancer cells

**Fig. 5:**
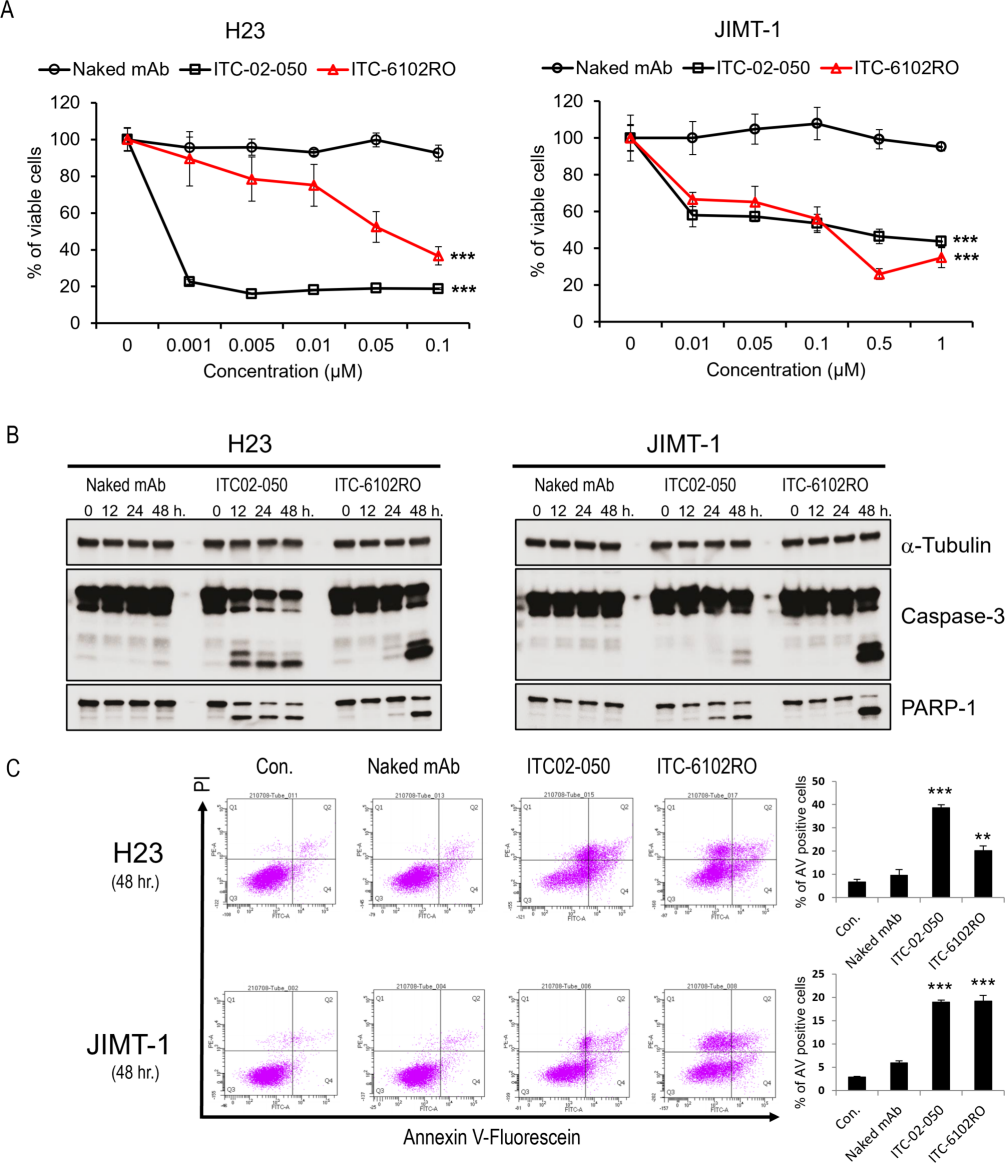
In vitro cytotoxic effects of ITC-6102RO through apoptosis. (**A**) H23 and JIMT-1 cells were treated with Naked mAb, ITC-02-050 (free drug), or ITC-6102RO at various concentrations (0, 0.001 ~ 0.1 µM) for 48 h. Each point represents the mean and SD (n = 8). After incubation, cellular viability was measured using the cell counting kit-8. Data are shown as mean ± standard deviation. ***p < 0.001 versus Naked mAb-treated cells. (**B**) Proteolytic processing of caspase-3 by ITC-6102RO. H23 and JIMT-1 cells were treated with Naked mAb, ITC-02-050 (free drug), or ITC-6102RO at 0.1-µM for the indicated times. Cell extracts were prepared for Western blotting to detect changes in the expression of caspase-3 and PARP-1. Equal loading of protein samples was verified using Western blotting of α-tubulin. (**C**) The apoptotic effects of ITC-6102RO in H23 and JIMT-1 cell lines treated with Naked mAb, ITC-02-050 (free drug), or ITC-6102RO at 0.1-µM for 48 h. Each cell line was stained using Annexin V-PI and examined by flow cytometry as described in the Materials and Methods. The results presented are mean ± SD, with n = 3 replicates in each group. **p < 0.01, ***p < 0.001 versus Naked mAb-treated cells

To assess the B7-H3-specific cytotoxicity of dHBD, including ITC-6102RO, human lung cancer H23 cells and human breast cancer JIMT-1 cells were treated with ITC-6102RO, and their viabilities were evaluated. A dose-dependent decrease in cell viability was observed in B7-H3-positive cancer cell lines treated with ITC-6102RO in vitro (Fig. 5A). No significant cytotoxic activity was detected in cell lines treated with Naked mAb. ITC-6102RO reduced the viability of H23 cells by 100, 89.5, 78.5, 75.1, 52.4, and 36.6% at concentrations of 0, 0.001, 0.005, 0.05, 0.1, and 0.1 µM, respectively, and decreased the viability of JIMT1 cells by 100, 66.6, 65.1, 56.0, 26.0, and 34.9% at concentrations of 0, 0.01, 0.05, 0.1, 0.5, and 1 µM, respectively. Notably, ITC-6102RO demonstrated comparable cell growth inhibition to ITC-02-050 (free drug) in the JIMT-1 cell line. These findings indicate that the cytotoxic potential of ITC-6102RO is retained following conjugation.

Subsequently, the involvement of apoptosis in the reduction of cell viability by ITC-6102RO was investigated. Classic apoptosis markers, including cleaved caspase-3 and cleaved-PARP, were observed at 48 h in H23 and JIMT-1 cells (Fig. 5B). Apoptosis was also assessed in treated and untreated cells using PI/Annexin V-FITC staining. The data revealed that upon treatment with control Naked mAb, ITC-02-050, and ITC-6102RO, the percentages of apoptotic cells were 6.7, 9.55, 38.55, and 20.15% in H23 cells, and 2.9, 5.9, 19, and 19.2% in JIMT-1 cells, respectively (Fig. 5C). These findings suggest that ITC-6102RO’s inhibitory effect on cell viability induces apoptosis through a caspase-3-dependent pathway.

### ITC-6102RO induced S phase arrest and DNA damage by dHBD (heterocycle-fused benzodiazepine dimer)

**Fig. 6:**
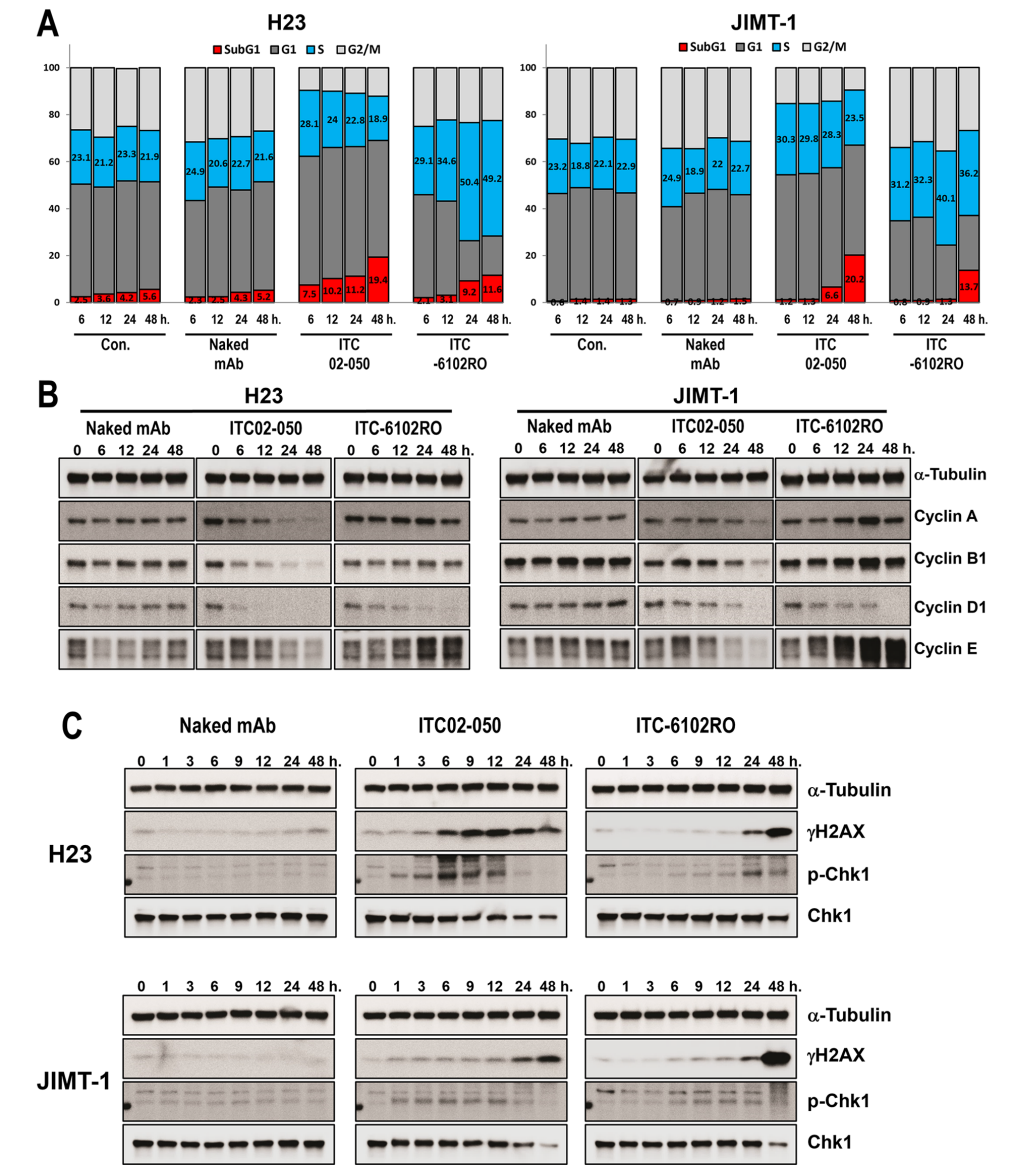
Disruption of cell cycle and DNA damage by ITC-6102RO. (**A**) Cell cycle distribution in H23 and JIMT-1 cells after treatment with Naked mAb, ITC-02-050 (free drug), or ITC-6102RO at 0.1 µM for 48 h. Propidium iodide (PI)-stained cells were analyzed using flow cytometry. (**B**) Cyclin expression changes in response to treatment with Naked mAb, ITC-02-050 (free drug), or ITC-6102RO. H23 and JIMT-1 cells were treated with Naked mAb, ITC-02-050, or ITC-6102RO at 0.1 µM for the indicated periods, and Western blotting was performed to detect expression changes of cyclins. (**C**) DNA damage effect induced by Naked mAb, ITC-02-050, or ITC-6102RO. H23 and JIMT-1 cells were treated with Naked mAb, ITC-02-050, or ITC-6102RO at 0.1 µM for the indicated periods, and Western blotting was conducted using γH2AX, p-Chk1, and Chk1 antibodies

The dHBD was modified to generate ITC-6102RO from the pyrrolobenzodiazepine (PBD) structure, enhancing its solubility and retaining its potent DNA alkylation properties akin to those of PBD. To investigate the effects of ITC-6102RO on cell cycle progression and DNA damage, H23 and JIMT-1 cells were treated with 0.1 µM of the compound, followed by DNA content analysis using flow cytometry and Western blotting for cyclins, gamma-H2AX, and phosphor-Chk1. At 24 h post-treatment, ITC-6102RO significantly increased the proportion of cells in the S phase, while Naked mAb did not alter the distribution of cells in different phases of the cell cycle (Fig. 6A and Additional file 1). Cyclin E levels also increased in a time-dependent manner with ITC-6102RO treatment compared to Naked mAb treatment (Fig. 6B), suggesting that the dHBD component of ITC-6102RO may cause S phase arrest. Furthermore, ITC-6102RO elevated gamma-H2AX and phosphorylated Chk1 levels after 24 h (Fig. 6C). A strong induction of the sub-G1 population was observed in H23 and JIMT-1 cells following S phase arrest at 48 h (Fig. 6A). Collectively, these findings demonstrate that ITC-6102RO induces apoptosis through cell cycle arrest in the S phase and DNA damage, which are attributable to the dHBD component of ITC-6102RO.

### ITC-6102RO demonstrates effective tumor growth inhibition in B7-H3-positive CDX and PDX models

**Fig. 7:**
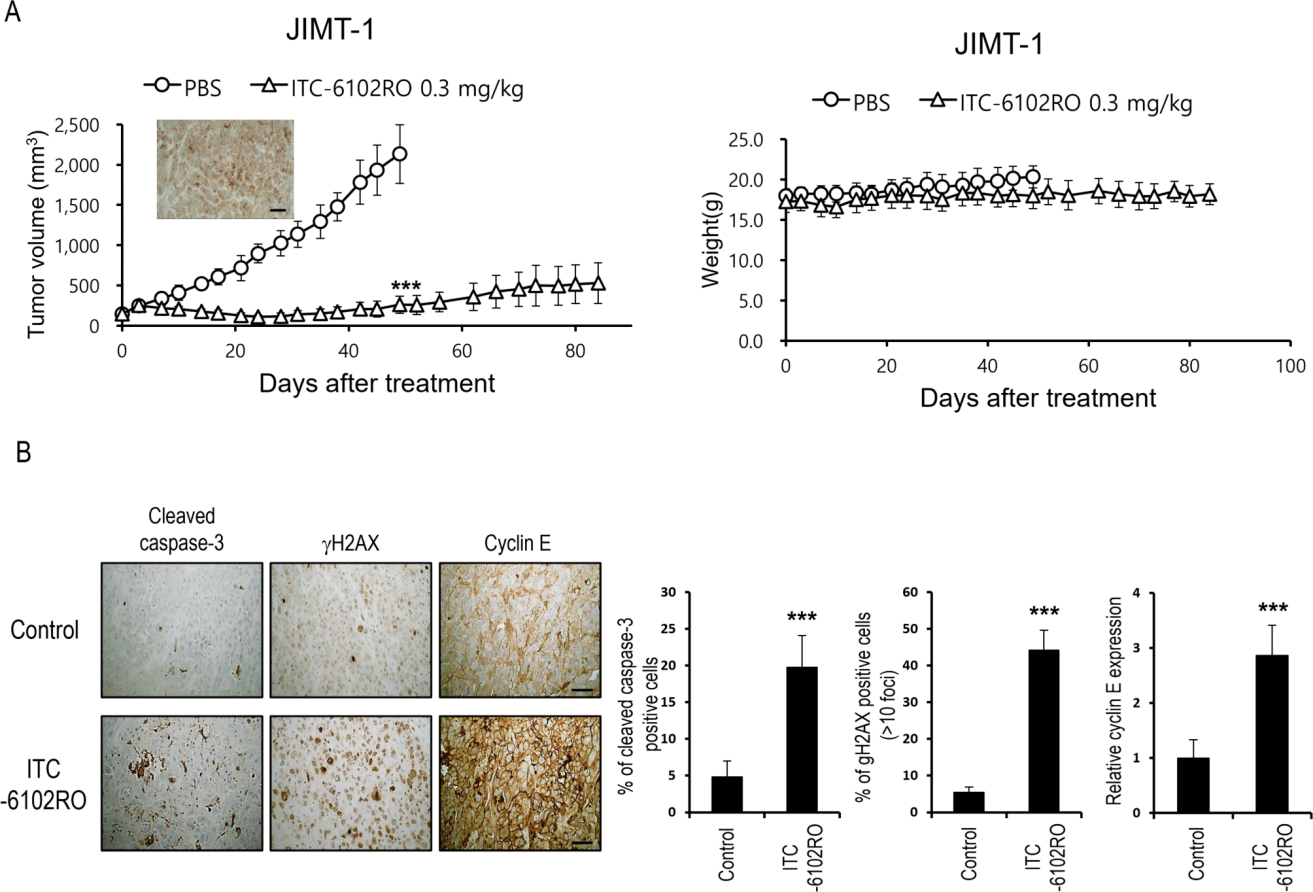
Efficacy of ITC-6102RO in the CDX model. (**A**) Therapeutic efficacy of ITC-6102RO in the JIMT-1 xenograft model. The drugs were intravenously injected at 0.3 mg/kg once, n = 5. B7-H3 expression in tumor tissue was detected using IHC (brown, DAB-stained B7-H3; blue, hematoxylin-stained nuclei; scale bar, 10 μm). (**B**) Mice bearing JIMT-1 tumors were treated intravenously with a single 10 mg/kg dose of ITC-6102RO. One day after treatment, tumors were harvested, embedded in paraffin, and sectioned. DAB images of immunohistochemically stained cleaved caspase-3, γH2AX, and cyclin E were obtained under a microscope. Scale bar, 10 μm. Data values represent the mean ± standard deviation. ***p < 0.001 versus control

To evaluate the antitumor efficacy of ITC-6102RO, we investigated its in vivo effects on tumor growth inhibition in mice bearing JIMT-1 xenograft tumors. B7-H3 expression in the JIMT-1 xenograft tumor was confirmed by immunohistochemistry (IHC; Fig. 7A). JIMT-1 tumor-bearing mice were intravenously treated with either PBS or a single dose of 0.3 mg/kg ITC-6102RO. Significant tumor growth inhibition was observed in the ITC-6102RO-treated group compared to the PBS-treated group (Fig. 7A). No substantial changes in body weight were detected throughout the experiment, indicating that the mice did not experience severe toxicity (Fig. 7A).

To further examine the pharmacological effects of ITC-6102RO in vivo, IHC staining of JIMT-1 tumor tissues was performed using antibodies against cleaved-caspase-3 (apoptosis), gamma-H2AX (DNA damage), and cyclin E (S phase checkpoint). Increased levels of apoptotic cells, DNA damage, and S phase arrest were observed following ITC-6102RO treatment, but not with PBS treatment (Fig. 7B). The percentages of apoptotic cells, DNA damage, and S phase arrest were significantly higher in the group treated with 0.3 mg/kg ITC-6102RO compared to the PBS-treated group (*p* < 0.001; Fig. 7B). These results suggest that the DNA alkylating agent, dHBD, was effectively delivered to JIMT-1 tumor cells by the anti-B7-H3 monoclonal antibody, leading to apoptotic cell death following DNA damage and S phase arrest.

**Fig. 8:**
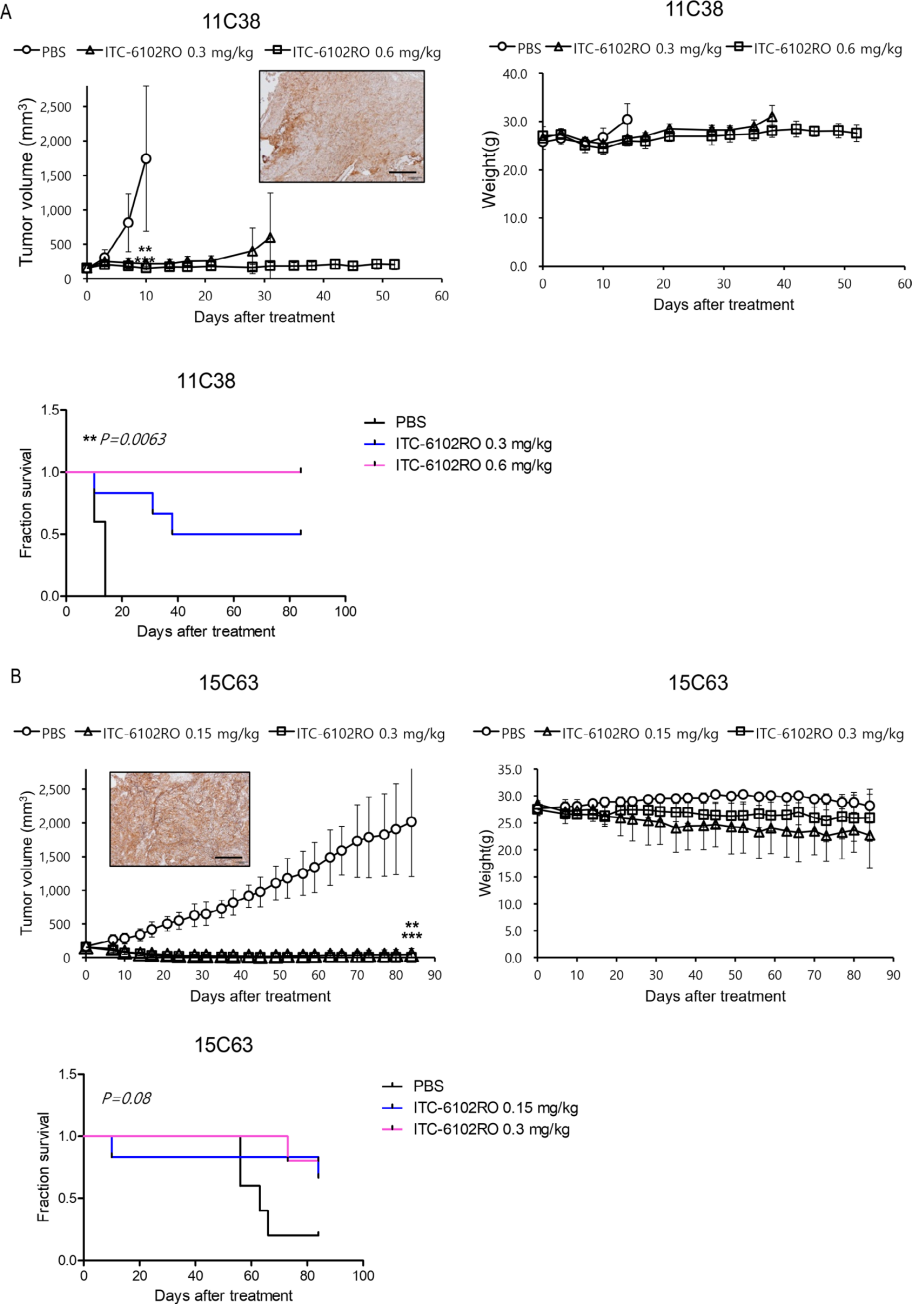
In vivo efficacy of ITC-6102RO in B7-H3-positive lung cancer PDX models. (**A**) Therapeutic efficacy of ITC-6102RO in 11C38 lung cancer PDX model. The drug was intravenously injected at 0.3 or 0.6 mg/kg, once, n = 5. (**B**) Therapeutic efficacy of ITC-6102RO in 15C63 lung cancer PDX models. The drug was intravenously injected at 0.15 or 0.3 mg/kg, once, n = 5. B7-H3 expression in tumor tissue was detected using IHC (brown, DAB-stained B7-H3; blue, hematoxylin-stained nuclei, scale bar, 50 μm). Data values represent the mean ± standard deviation. ***p < 0.001

Subsequently, the clinical applicability of ITC-6102RO was evaluated through its in vivo efficacy in PDX tumor models, which are considered the most relevant models for patients. PDX models, generated from B7-H3 highly-expressing human lung tissue, were subjected to ITC-6102RO treatment (Fig. 8A, B). In the 11C38 model, tumor growth was suppressed by 95.9% and 100.1% with ITC-6102RO doses of 0.3 mg/kg and 0.6 mg/kg, respectively, on day ten, accompanied by a significantly increased survival rate (Fig. 8A). In the 15C63 PDX model, tumor growth was inhibited by 106% with ITC-6102RO treatments at 0.15 mg/kg, and by 108.5% with treatments at 0.3 mg/kg. Tumor-free CR was observed in all mice within the 0.3 mg/kg ITC-6102RO-treated group at the endpoint (Fig. 8B). Moreover, three out of five mice were tumor-free in the 0.15 mg/kg ITC-6102RO-treated group at the endpoint, displaying a moderately enhanced survival rate. These findings unequivocally demonstrate the clinical potential of the B7-H3-targeting ITC-6102RO, developed using our OHPAS linker-toxin technology, as a potent novel B7-H3 ADC in conjunction with existing B7-H3-targeting therapeutics.

## Discussion

ADCs combine the specificity of an antibody with the cytotoxicity of conventional chemotherapy agents, emerging as a prominent anticancer therapy in recent clinical successes [27]. These conjugates exhibit a broad therapeutic window compared to traditional cancer chemotherapeutic agents [28]. ADCs leverage the specificity of antibodies to target and internalize toxic payloads into tumor cells [21, 29]. Subsequently, antigens bound to the ADC antibody are transported to lysosomes, where ADCs undergo internalization and toxic payloads are released into tumor cells through endocytosis [30]. The released cytotoxic drugs induce target cell death via DNA damage or microtubule inhibition. Despite the apparent anticancer potential of ADCs, designing an optimal ADC with the appropriate antibody, linker, and small molecule payload (toxin) remains challenging, with limited commercially available options.

Histological overexpression of B7-H3 has been observed in various cancers and is closely linked to immune surveillance and tumor progression mediated by B7-H3 [12, 31–33]. Several B7-H3-targeting treatment modalities, including antibody-based cytotoxic therapies, CD3 bispecific antibody anticancer agents, chimeric antigen receptor (CAR)-T cell therapies, and ADCs, are under development [34–36]; however, no B7-H3-targeting therapeutic agents have been approved thus far.

In this study, we generated ITC-6102RO, a novel B7-H3-ADC with an OHPAS linker, which demonstrated biostability in mouse and human plasma and exhibited B7-H3-specific antitumor activity in preclinical models of solid tumor types. In vitro studies revealed that ITC-6102RO induced potent anticancer activity via DNA damage and apoptosis in B7-H3-overexpressing cell lines, while the anti-B7-H3 monoclonal antibody (Naked mAb) did not exhibit anticancer activity in any B7-H3-overexpressing cell lines (Figs. 5 and 6). ITC-6102RO was found to induce S phase arrest in the cell cycle through the internalization of dHBD into cancer cells (Figs. 3 and 6). In vivo studies showed that ITC-6102RO significantly inhibited tumor growth in B7-H3-overexpressing JIMT-1 xenograft models and in two cases of B7-H3-overexpressing lung cancer PDX models (Figs. 7 and 8). Consistent with in vitro results, S phase arrest (cyclin E accumulation), upregulated DNA damage (gamma-H2AX foci), and apoptotic cells (cleaved caspase-3) were observed in JIMT-1 tumor tissues in vivo at the endpoint (Fig. 7).

The ADC, ITC-6102RO, selectively internalizes into cancer cells expressing B7-H3 and subsequently induces the release of dHBD upon transportation to the lysosome (Fig. 3B). However, for all targeted therapies, establishing guidelines for target expression is crucial. Therefore, determining a criterion for B7-H3 expression is a prerequisite for utilizing ITC-6102RO as a therapeutic agent, and this is currently being investigated.

The OHPAS linker in ITC-6102RO exhibited high stability in both mouse and human plasma. Moreover, the payload used for ADC construction, dHBD, demonstrated effective anticancer efficacy in the form of HBD, which enhanced its solubility. Notably, there was a weight loss issue in the lung cancer PDX mouse model; however, it was not severe, and further clarification of safety profiles is required. The pharmacokinetic profiles and stability of ITC-6102RO in rat plasma were comparable to those of the anti-B7-H3 monoclonal antibody, as evidenced by the in vivo analysis (Fig. 4). Furthermore, since B7-H3 is known to modulate T cell function in both costimulatory and coinhibitory manners, the potential for combination therapy involving ITC-6102RO and immunotherapy merits future consideration.

## Conclusion

In summary, the study has successfully generated ITC-6102RO, a novel B7-H3-targeted ADC utilizing OHPAS-linker technology. The preclinical data demonstrates its potent antitumor activity and acceptable safety profile across various models. These findings indicate that ITC-6102RO holds promise as a potential therapeutic strategy for patients with B7-H3-overexpressing solid tumors. Notably, ITC-6102RO exhibited remarkable anticancer efficacy, as evidenced by its inhibition of tumor growth and enhancement of survival rates after a single treatment. Consequently, this technology signifies a powerful new ADC platform with potential applicability for a range of targets.

### Electronic supplementary material

Below is the link to the electronic supplementary material.

Supplementary Material 1

## Data Availability

Not applicable.

## List of Abbreviations

ADC: Antibody-drug conjugate
dHBD: Heterocycle-fused benzodiazepine dimer
OHPAS: Ortho Hydroxy-Protected Aryl Sulfate
PBD: Pyrrolobenzodiazepine
PDX: Patient-derived xenograft
VC-PABC: Valine-citrulline-*p*-aminobenzyloxycarbonyl
