# Supplementary material for: ITC-6102RO, a novel B7-H3 antibody-drug conjugate, exhibits potent therapeutic effects against B7-H3 expressing solid tumors
Source: Cancer Cell Int. 2023 Aug 18;23:172. doi: 10.1186/s12935-023-02991-x (PMC10439577; doi:10.1186/s12935-023-02991-x)
Supplement: Supplementary file 1 — Supplementary Material 1 [file 12935_2023_2991_MOESM1_ESM.pdf]

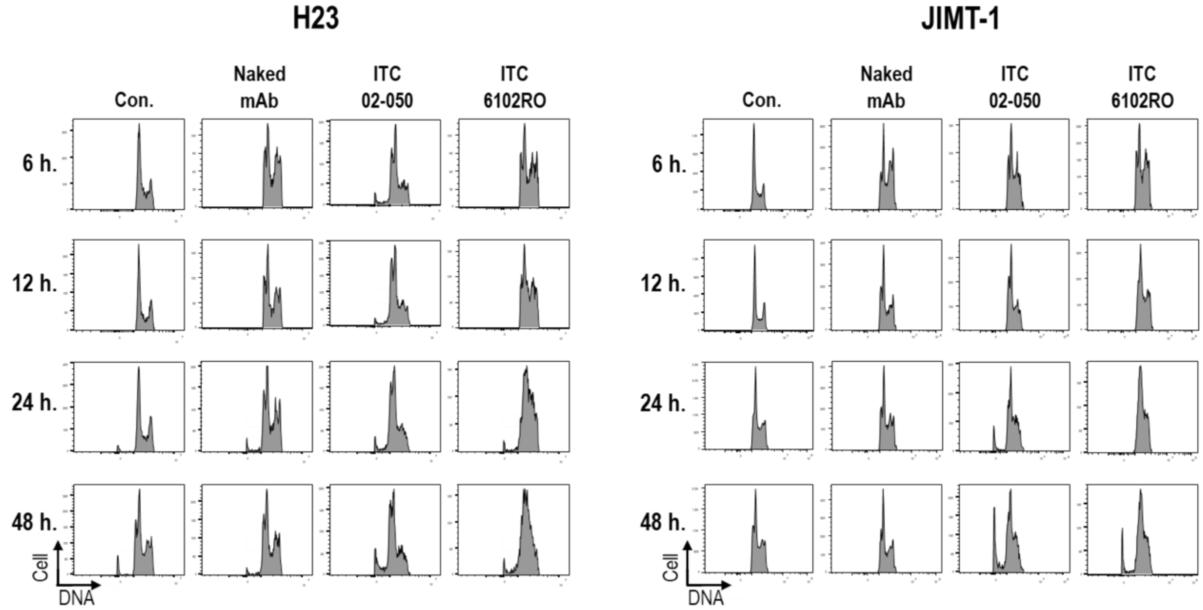


**Additional file 1. Histogram of cell cycle and DNA damage by ITC-6102RO**

A. Histogram of cell cycle distribution in H23 and JIMT-1 cells after treatment with Naked mAb, ITC-02-050 (free drug), or ITC-6102RO at 0.1 μM for 48 h. Propidium iodide (PI)-stained cells were analyzed using flow cytometry.
